# MiR-363 restrain the proliferation, migration and invasion of colorectal carcinoma cell by targeting E2F3

**DOI:** 10.7150/jca.83897

**Published:** 2023-05-15

**Authors:** Wenyu Yang, Xiqian Yang, Yang Zhang, Yunhai Li, Wenliang Lv

**Affiliations:** Clinical College of Chinese Medicine, Hubei University of Chinese Medicine, Wuhan 430061, China.

**Keywords:** miR-363, E2F3, colorectal cancer, migration, invasion

## Abstract

MicroRNA (miRNA) is associated with tumor cell proliferation, migration and invasion. Studies have shown that miRNAs are closely related to the occurrence and development of colorectal cancer (CRC), but the mechanisms deserve further investigation. In this study, we aim to explore the role of miR-363 on CRC tumorigenesis. Using CRC cell lines, we tested the expression of miR-363 by using RT-PCR, and miR-363 effect on cell behavior was test by using CCK-8 assay, wound-healing assay and cell invasion assay, and western blotting. Luciferase reporter assay and western blot confirmed that E2F3 was the target gene for miR-363. We further examined the effect of E2F3 on the regulation of miR-363 on cell behavior through knockdown of E2F3. Western blot and RT-PCR assay showed that miR-363 inhibited the expression of E2F3 in HCT-116 and SW480 cell. MiR-363 overexpression or E2F3 knockdown inhibited cell proliferation, migration and invasion of CRC. This study demonstrated that miR-363 is able to suppress cell proliferation, migration and invasion by negative regulating E2F3 in CRC cells, and inhibits tumor growth *in vivo*.

## Introduction

Colorectal cancer (CRC) is one of the most common gastrointestinal malignancies clinically. According to the study released by WHO's International Agency for Research on Cancer in 2020, CRC has become the third most common cancer after breast cancer and lung cancer. In China, the incidence and mortality of CRC are on the rise 1. Up to now, China has the highest morbidity and mortality of CRC in the world, and the 5-year survival rate of CRC patients is only 31% 2. Thus, it is of great significance to clarify the pathogenesis of CRC and find potential biomarkers for better CRC clinical treatment.

MicroRNAs (miRNAs) are small non-coding RNAs that play an important role in regulating post-transcriptional gene expression 3, 4. MiRNAs decreased the protein translation or mRNA degradation through binding 3'UTR of their target genes 5, 6. Aberrant expression of miRNAs is associated with the development of CRC 7-9. MiR-362, which down-regulated in CRC, suppressed cell proliferation, migration and invasion by targeting SIX1 10. MiR-17 promotes cell proliferation and migration in human colorectal cancer by downregulating SIK1 11. MiRNA-363 is abnormally expressed in a variety of tumors, and regulates cell proliferation and apoptosis by regulating different target genes 12-15. MiRNA-363 can affect the hepatoma cell proliferation by inhibiting the expression of HMGA2 16. Reduced miR-363 expression attenuates the invasion and migration of neuroblastoma cells 17. Studies have shown that inhibiting the expression of miR-363 can promote lymph node metastasis of head and neck squamous cell carcinoma 18. Paulína Pidíkova et al. observed that miRNAs (miR-1, miR-15, miR-16, miR-133, miR-143, miR-192, miR-195, miR-206, miR-367 and miR-497) were down-regulated in CRC, and its high expression was usually related to better clinical survival rate, suggesting that down-regulated miRNAs in CRC may be potential targets for the CRC treatment 19. Yan et al. observed that miR-363 was underexpressed in CRC, and overexpression of miR-363 could inhibit the proliferation and migration of CRC cells 20. Hu et al. observed that overexpression of miR-363 inhibited cell migration, invasion and EMT by negatively regulating Sox4 21. Overexpression of miR-363 in HT29 and HCT116 cell lines effectively inhibited cell proliferation and metastasis, and promoted apoptosis, and the mechanism is by targeting SphK2 22. Since the intermolecular regulation in human body is very complex, a molecule can be regulated by a variety of other molecules, or can be combined to regulate many molecules, the regulation between molecules is not one-to-one correspondence, and the occurrence and development of diseases also exist multiple targets and multiple pathways. MiR-196 can promote or suppress cancer by targeting genes such as GATA6, SOCS1, SOCS3, ANXA1, DFFA, PDCD4, ZG16 and ING5 23. Thus, miR-363 may bind to a variety of downstream molecules and further exert inhibitory effects. Although progress has been made in the research on the mechanism of miR-363 in the treatment of CRC, it is still of great significance to further clarify it.

The E2F family was first discovered in the E2 gene of adenovirus, and a total of eight E2F members have been discovered. Due to the differences in structure, the functions of different members are not the same 24. E2F3 is involved in the regulation of G1/S cell cycle and affects the rate of DNA synthesis, which is closely related to the occurrence of liver cancer, breast cancer, colon cancer, etc. 25, and is well known as oncogenes in cancer initiation and progression 26. Clinical studies have found that the higher the clinical stage and pathological grade of the tumor, the higher the positive rate of E2F3 expression 27. Olsson et al. also conducted a study on bladder cancer and prostate cancer cells, and the results showed that overexpression of E2F3 in bladder cancer cells significantly improved the proliferation ability of tumor cells 26. MiR-128 inhibited CRC proliferation and metastasis through E2F3 28. MiR-503 inhibits cell proliferation and induces apoptosis in colorectal cancer cells by targeting E2F3 29. Jiang et al. showed that miR-363 can target the expression of E2F3 and inhibit the proliferation and invasion of HepG2 cells 30, but whether miR-363 can play its role by inhibiting the expression of E2F3 in CRC cells has not been reported, and further studies are needed.

In this study, we demonstrated that miR-363 was down-regulated in CRC, and miR-363 overexpression significantly inhibited CRC cell proliferation, migration and invasion, and this effect may be achieved by inhibiting E2F3 expression.

## Materials and methods

### Cell culture and transfection

The HCT-116, SW480 human CRC cell lines and the human normal colonic epithelial cell line NCM460 were purchased from Shanghai Cell Bank, Chinese Academy of Sciences. HCT-116 and NCM460 cells were maintained in McCoy's 5A medium (Gibco, Thermo Fisher Scientific, Inc.) supplemented with 10% fetal bovine serum (FBS, Gibco, Thermo Fisher Scientific, Inc.), SW480 cells were cultured in Dulbecco modified Eagle medium (DMEM) supplemented with 10% FBS, and all cells were incubated at 37℃ in a 5% CO2 incubator. Cells were seeded in six-well plates and transfected with miR-363 mimics, E2F3-siRNA and negative control using lipofectamine 2000 (Invitrogen, Carlsbad, CA, USA) following the manufacturer's protocol. The sequences of miR-363 mimics were: 5'-AAUUGCACGGUAUCCAUCUGUA-3', and the sequences of negative control were: 5'-UUCUCCGAACGUGUCACGU-3'.

### RT-PCR analysis

Cells were lysed in Trizol (Invitrogen, Carlsbad, CA, USA) for total RNA extraction using reverse transcriptase kit (TAKARA, USA). qRT-PCR was performed using a real-time system (Bio-Rad) using the SYBR Green PCR Kit (KM4101, KAPA Biosystems). The results analysis by 2^-△△Ct^ method, and the miR-363 expression was normalized to the endogenous expression of U6 small nuclear RNA. The mRNA expression level of E2F3 normalized to the endogenous expression of GAPDH. All primers were designed and produced by Nanjing Kingsy Biotechnology Co., Ltd. and are summarized in Table 1.

### Luciferase reporter assay

Targetscan (www.targetscan.org) bioinformatics software was utilized to predict the potential target genes of miR-363. The result showed that there were seven base binding sites between miR-363 and E2F3 3'-UTR. The putative target sites of the human E2F3 3ʹ-UTR segments for miR-363 were amplified by PCR. 5 × 10^5^ cells were seeded in 24-well plates and co-transfected with E2F3 3ʹ-UTR (0.25 µg) or mutant E2F3 3ʹ-UTR and miR-363 mimics (50 nM) or NC using lipofectamine 2000. The activities of firefly and Renilla luciferase were detected using the dual-luciferase reporter assay system (Genecopoeia, USA) and evaluated by SynergyH multiscan spectrum (Bio-Tek, USA).

### Cell proliferation

Cell proliferation was identified using CCK8 assay performed as described 31. 24 hours later with transfection, cells were seeded into 96-well plates (5×10^3^ cells/well). CCK-8 solution (10 μl) was added to each well, and after 4 h, the absorbance was measured at 450 nm using Multiskan FC (Thermo Fisher, USA).

### Colony formation assay

To assess colony formation, 500 cells were seeded in each well of a 6-well plate and maintained in medium containing 10% FBS at 37°C. The medium was replaced every three days. After 14 days, colonies were fixed with methanol and stained with 0.1% crystal violet (Sigma-Aldrich, Milwaukee, USA). Visible colonies were manually counted and triplicate measurements were acquired for each treatment group.

### Wound-healing assay

Cells were seeded in 6-well plates at 5 × 10^5^ cells per well. Before cell seeding, a line was drawn at the bottom of the well plate every 0.5 cm using a marker, and four lines were drawn in each well. On the next day, a scratch was made perpendicular to the well using a pipette tip against a ruler. The cells were washed with PBS and culture medium was added to the wells. Photographs were taken at 24 h to observe scratch healing. Image J software was used to measure the migration distances and calculate the cell migration ability.

### Cell invasion assay

For cell invasion assay, the upper Transwell chambers were pre-coated with 80 µl of Matrigel (354230; BD Biosciences, USA). The chambers were incubated at 37 °C for 30 minutes for gel formation and hydrated in FBS for 4 h before use. In the lower chambers, 600 µl of DMEM or McCoy's 5A medium containing 10% FBS was added. Then, cells were added to the upper chambers at a density of 1 × 10^5^ cells/well and incubated for 24 h at 4 °C. Subsequently, count the invaded cells under a microscope.

### Flow cytometry

Cells in each group were cultured for 24 h and then harvested, added in 1ml pre-cooled PBS, and centrifuge at 1000g, cell cycle and apoptosis of HCT-116 and SW480 cells were analyzed using flow cytometry according to the manufacturer's instructions and the data were analyzed by flow cytometry (Beckman Coulter, USA).

### Western blotting

Cells were washed with PBS and subjected to a lysis buffer. Protein lysates were separated using 15% SDS-PAGE and transferred to PVDF membranes. The membranes were blocked with a buffer containing 10% non-fat milk in PBS with 0.05% Tween-20 for 2 h and incubated with E2F3 antibody (1:1000, ab152126, Abcam), anti MMP-2 antibody (1:1000, ab97779, Abcam) and β-actin (1:1000, PAB36265, Bioswamp). Then the membranes were incubated with specific secondary antibodies attached to horseradish peroxidase at 4°C. Evaluation of the expression of proteins was performed using Image J version 1.38.

### Tumor formation assay in a nude mouse model

To validate the results *in vitro*, the tumorigenic ability of miR-363 was investigated *in vivo*. Immunodeficient BALB/C nu/nu male mice (n = 10; 5 weeks) are obtained from the Hubei Laboratory Animal Centre (Wuhan, China). All animals used in this study were strictly conformed to the international health and medical research guidelines for animal welfare and approved by the animal ethics committee of Wuhan Myhalic Biotechnological Co., Ltd (HLK-20210501-01). To evaluate the role of miR-363 *in vivo*, the stable overexpressed miR-363 CRC cells line was constructed by recombinant retrovirus at first. CRC cells of overexpressed miR-363 (miR-363-mimics), NC (negative control) and C (control) were respectively injected into the left and right forelimb of nude mice by subcutaneous. The tumor volume (V) was calculated according to the formula: length × (width^2^) / 2. The tumor formation in mice was monitored by the calipers every 3 days. Nude mice were euthanized with sodium pentobarbital (150 mg/kg, IP) at the experimental end-point, and then the tumors were harvested. When the mice rapidly lost 15-20% of their original body weight, could not feed themselves, or the tumor grew more than 10% of the animal's original weight, and other conditions caused extreme pain to the mice, the mice were euthanized after rigorous "Cost/Benefit Analysis".

### Statistical analysis

All statistical analysis was performed using SPSS 19.0 software (IBM Corp., Armonk). One-way analysis of variance followed by the Tukey's post hoc test was performed to compare differences between multiple groups. P < 0.05 was considered to indicate a statistically significant difference.

## Results

### MiR-363 was significantly down-regulated in CRC cell lines

**Figure 1:**
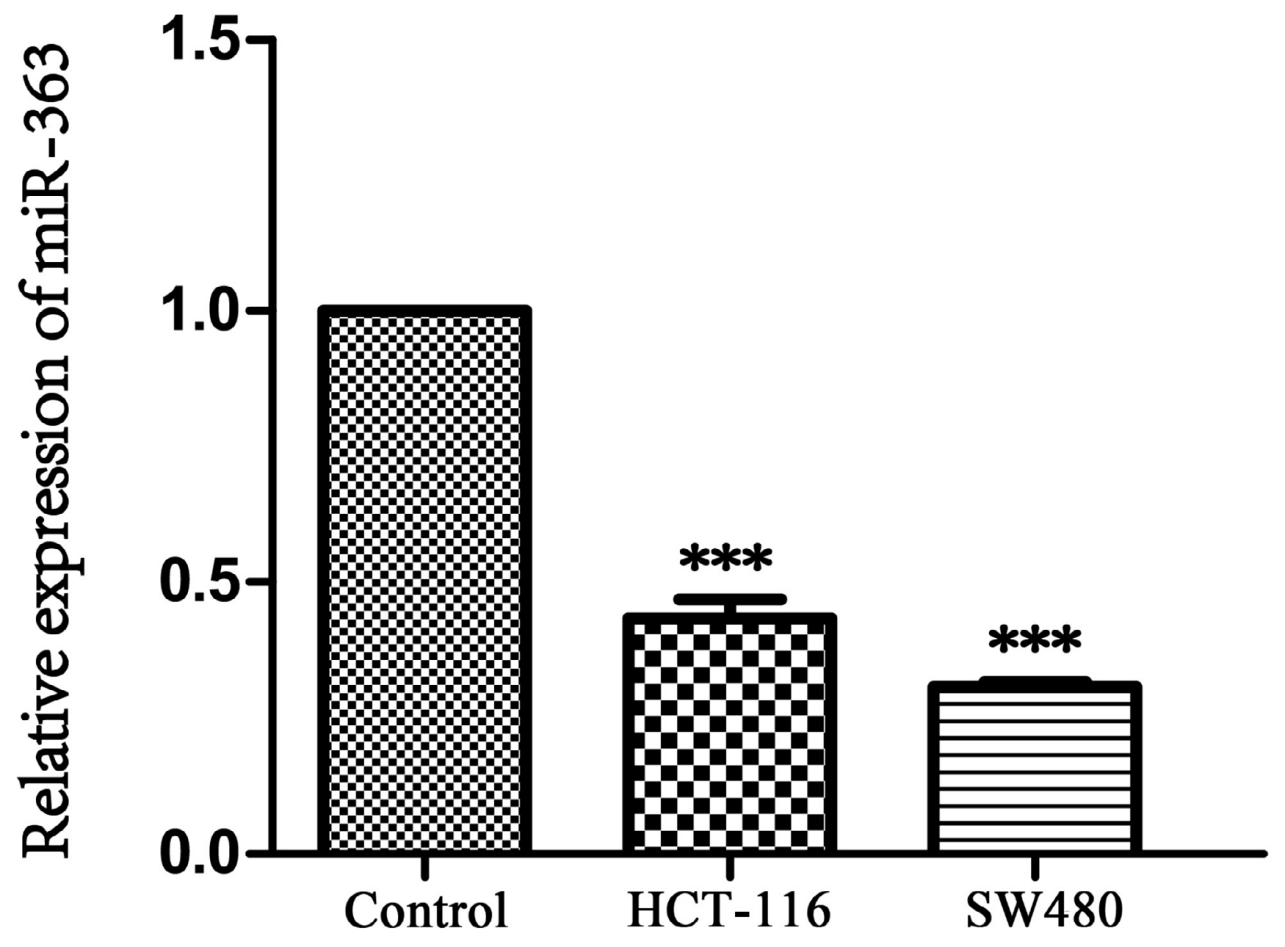
** Expression of miR-363 in human CRC cell lines.** The expression of miR-363 were detected in human CRC cell lines (HCT-116 and SW480) and the normal cell line (NCM460) by RT-PCR assay. n = 3. ^***^P<0.001. Take the average of normal group or C group as 1.

As the result showed in Figure 1, expression of miR-363 in HCT-116 and SW480 was significantly decreased compared with NCM460 (P < 0.001), indicating that miR-363 was significantly down-regulated in CRC cells.

### Overexpression of miR-363 inhibited cell proliferation, migration and invasion in CRC cells

**Figure 2:**
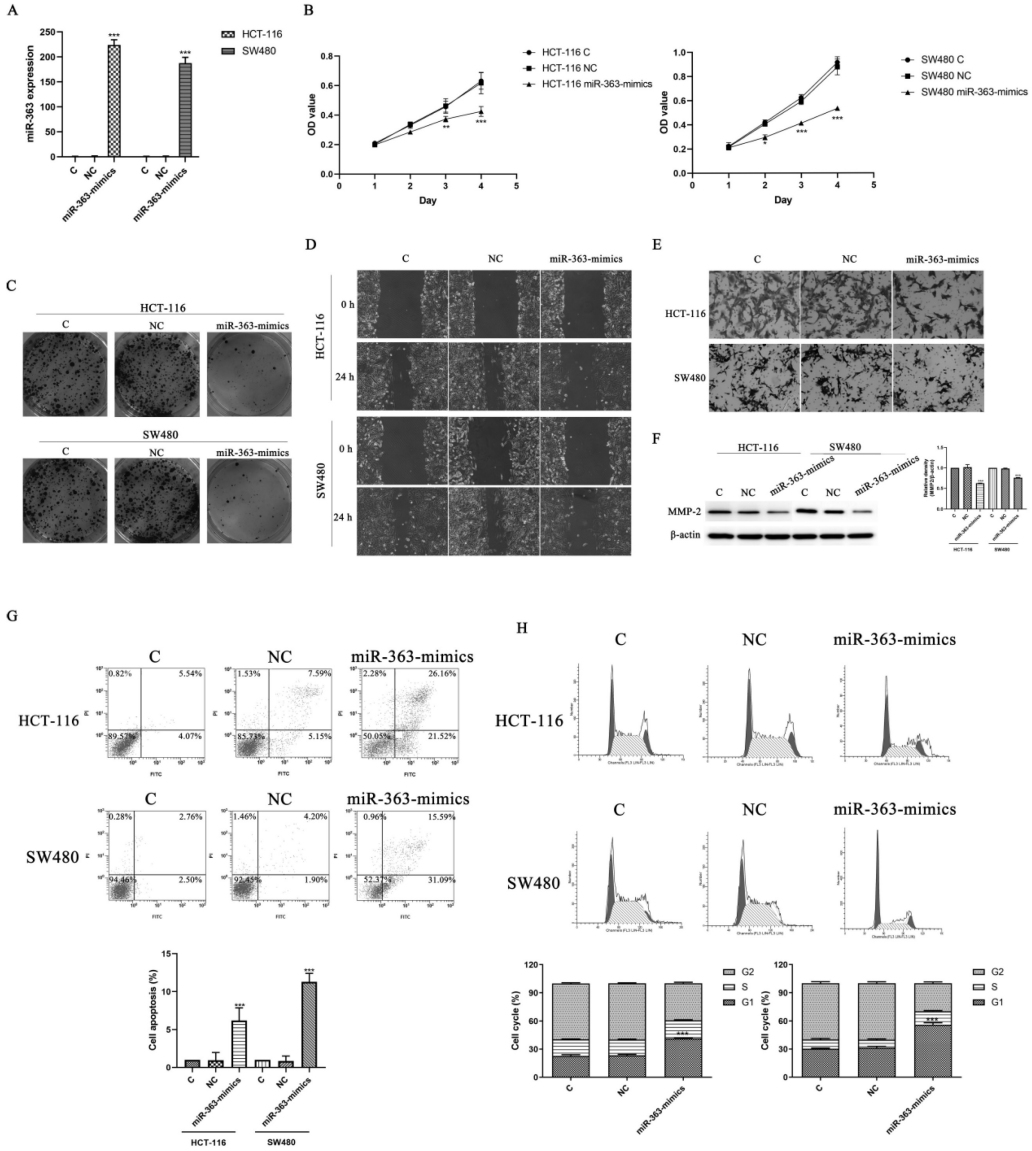
** Effect of miR-363 on CRC cell proliferation, migration and invasion.** A. miR-363 expression was determined by RT-PCR. B. CCK8 was used to measure the cell proliferation. C. Colony formation assays was performed to measure the cell proliferation. D. Wound-healing assay and E. cell invasion assay were used to test cell migration (100×) and invasion (200×). F. MMP-2 expression was detected by western blot. G. Cell apoptosis and H. cell cycle were detected by flow cytometry. n = 3. ^*^P<0.05,^ **^P<0.01, ^***^P<0.001. Take the average of normal group or C group as 1.

To explore the biological role of miR-363, the HCT-116 and SW480 cell were transfected with miR-363 mimics and negative control, and detected by using RT-PCR analysis (Figure 2A). The transfected HCT-116 and SW480 cells were tested for cell proliferation using CCK-8 assay. Compared with the negative control, the cell proliferation in miR-363 mimics group was significantly decreased (P<0.05, P<0.01, P<0.001) (Figure 2B), and colony formation assay exhibited that cell colony number was decreased in miR-363 mimics group compared with NC group (Figure 2C). Thus, miR-363 overexpression inhibited HCT-116 and SW480 cell proliferation.

Next, we studied the effects of miR-363 on cell migration and invasion in the HCT-116 and SW480 cell by using wound-healing assay and cell invasion assay. The results indicated that miR-363 overexpression inhibited the cell migration and invasion in the HCT-116 and SW480 cell compared to the negative controls (Figure 2D and 2E). Moreover, overexpression of miR-363 significantly reduced the expression level of invasion-related protein MMP-2 (P < 0.001) (Figure 2F). We further detected the effect of overexpression of miR-363 on apoptosis and cell cycle. As shown in Figure 2G and 2H, compared with the C and NC group, the apoptosis rate of miR-363-mimics group was significantly increased (P < 0.001), and G1 phase was significantly blocked (P < 0.001). Thus, miR-363 overexpression inhibited the CRC cell proliferation, migration and invasion, and promoted cell apoptosis.

### E2F3 was a target of miR-363 in CRC cell lines

**Figure 3:**
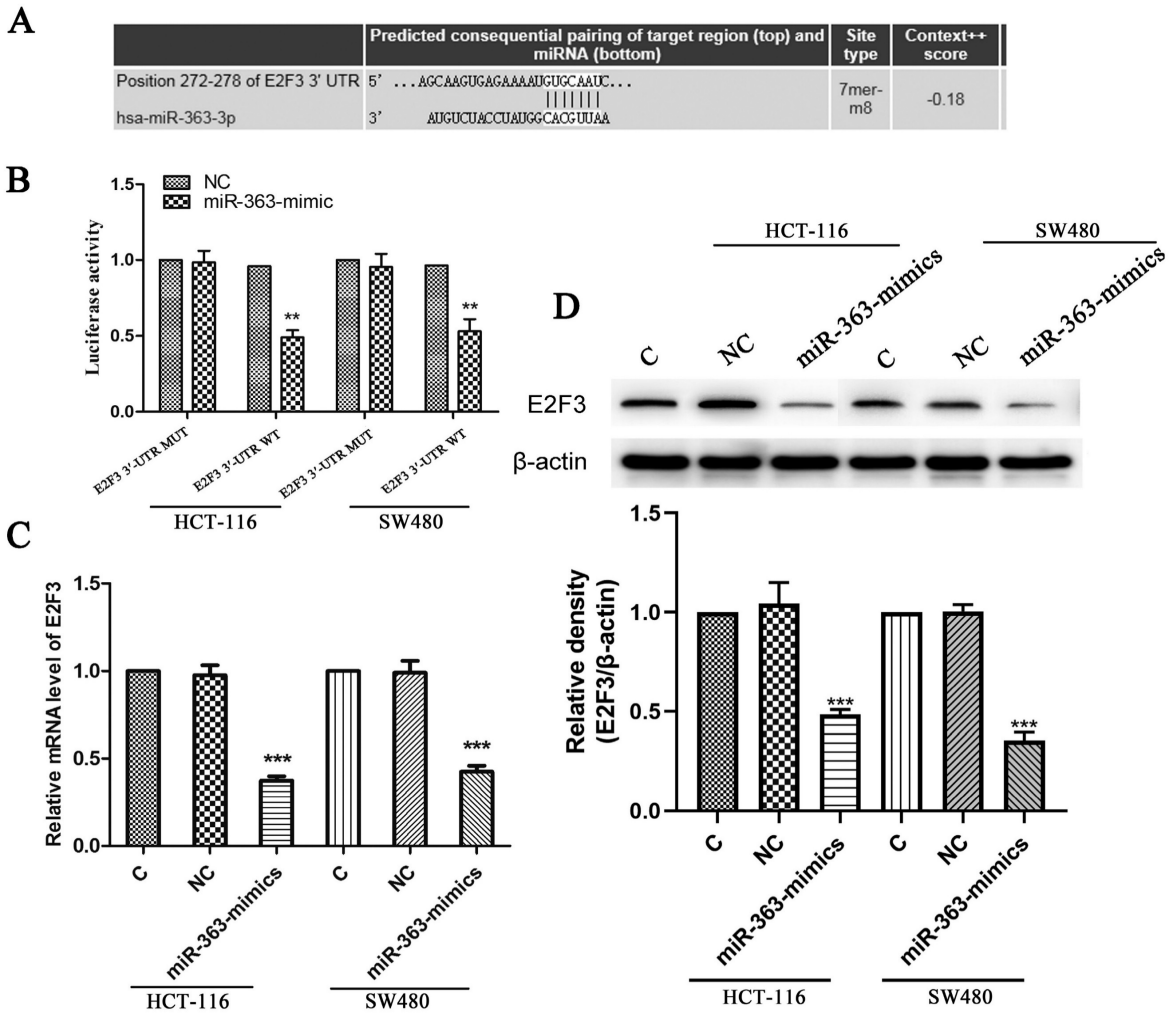
** E2F3 was the target of miR-363 in CRC cell lines.** The TargetScan database showed that miR-363 may bind to target sequences located at nucleotides 272-278 of E2F3 3'-UTR. B. Luciferase activity of E2F3 3'-UTR and mutant E2F3 3'-UTR were transfected with miR-363 mimics was detected by dual-luciferase reporter assay system. C. The mRNA levels of E2F3 were quantified by RT-PCR. D. E2F3 expression was measured by western blot. n = 3. ^***^P<0.001. Take the average of normal group or C group as 1.

The target genes of miR-363 were searched using the TargetScan (Figure 3A). We use luciferase reporter assay to pinpoint whether E2F3 is a direct target of miR-363. The results showed that luciferase activity in the group co-transfected with miR-363 mimics and E2F3 3ʹ-UTR wild type was significantly decreased (P<0.01) (Figure 3B). This result suggested that E2F3 is miR-363's target. Next, the Figure 3C shows that the levels of E2F3 in the miR-363-mimics group was markedly decreased (P < 0.001) (Figure 3C). WB detection results showed the same trend as before (Figure 3D).

### Knockdown of E2F3 significantly inhibited HCT-116 and SW480 cell proliferation, migration and invasion

**Figure 4:**
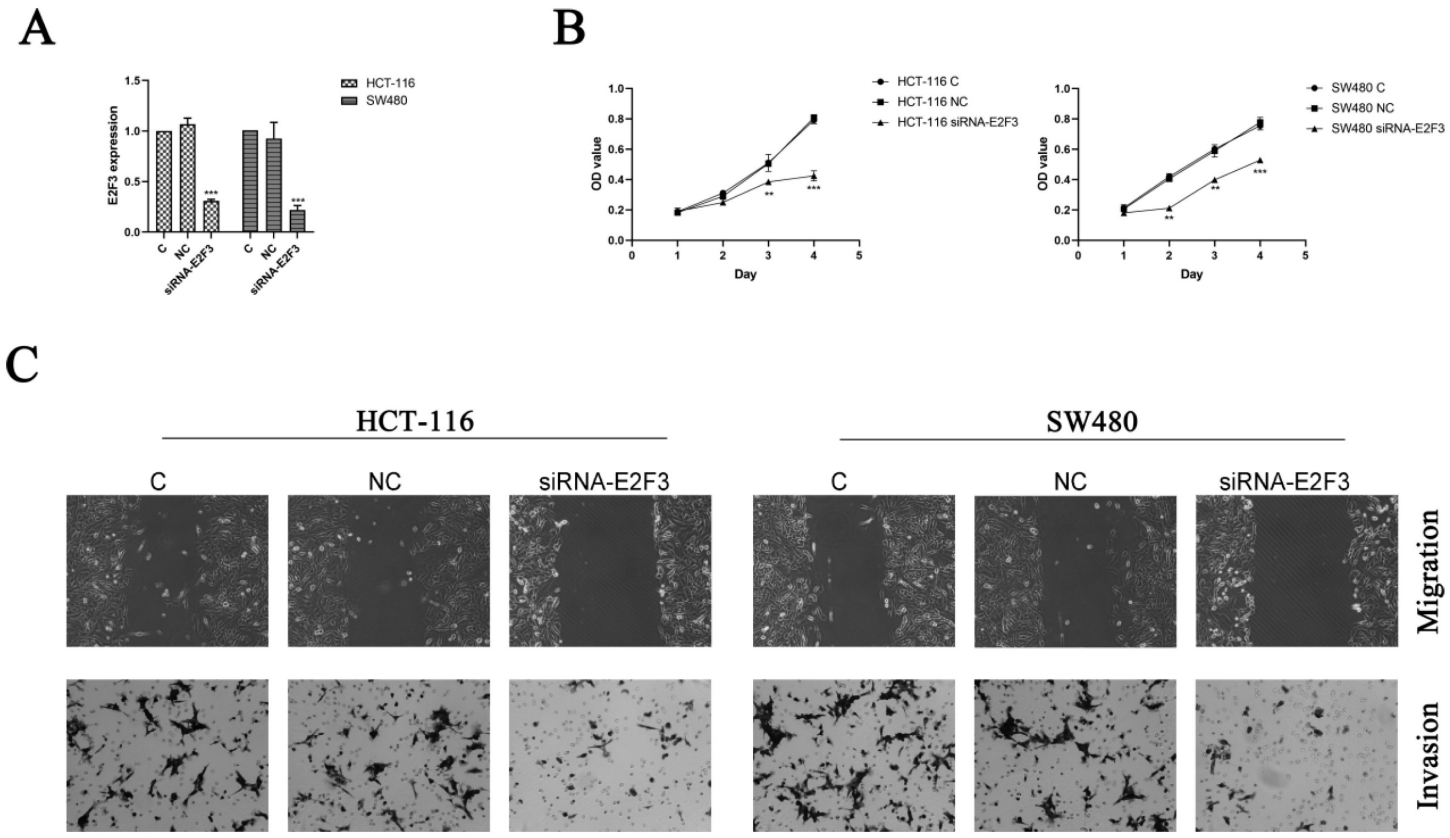
** Knockdown of E2F3 significantly inhibited HCT-116 and SW480 cell proliferation, migration and invasion.** A. Expression of E2F3 was determined by RT-PCR. B. Cell proliferation was test by CCK8. C. Cell migration and invasion were detected using wound-healing assay (100×) and cell invasion assay (200×). n = 3. ^*^P<0.05, ^**^P<0.01, ^***^P<0.001. Take the average of normal group or C group as 1.

To further confirm the hypothesis that overexpression of miR-363 suppresses cell behavior by targeting E2F3 in CRC, HCT-116 and SW480 cells were transfected with siRNA-E2F3 and negative control and observed by using RT-PCR analysis (Figure 4A). The transfected HCT-116 and SW480 cells were detected for cell proliferation by CCK8. Compared with the negative control, the cell proliferation in E2F3 knockdown group was significantly decreased (P < 0.01, P < 0.001) (Figure 4B).

E2F3 effects on cell migration and invasion in the HCT-116 and SW480 cells were observed by using wound-healing assay and cell invasion assay, and the results indicated knockdown of E2F3 restrained the cell migration and invasion in the HCT-116 and SW480 cells compared to the NC group (Figure 4C). Thus, miR-363 suppresses cell behavior by targeting E2F3 in CRC cells.

### MiR-363 suppressed tumor growth *in vivo*

**Figure 5:**
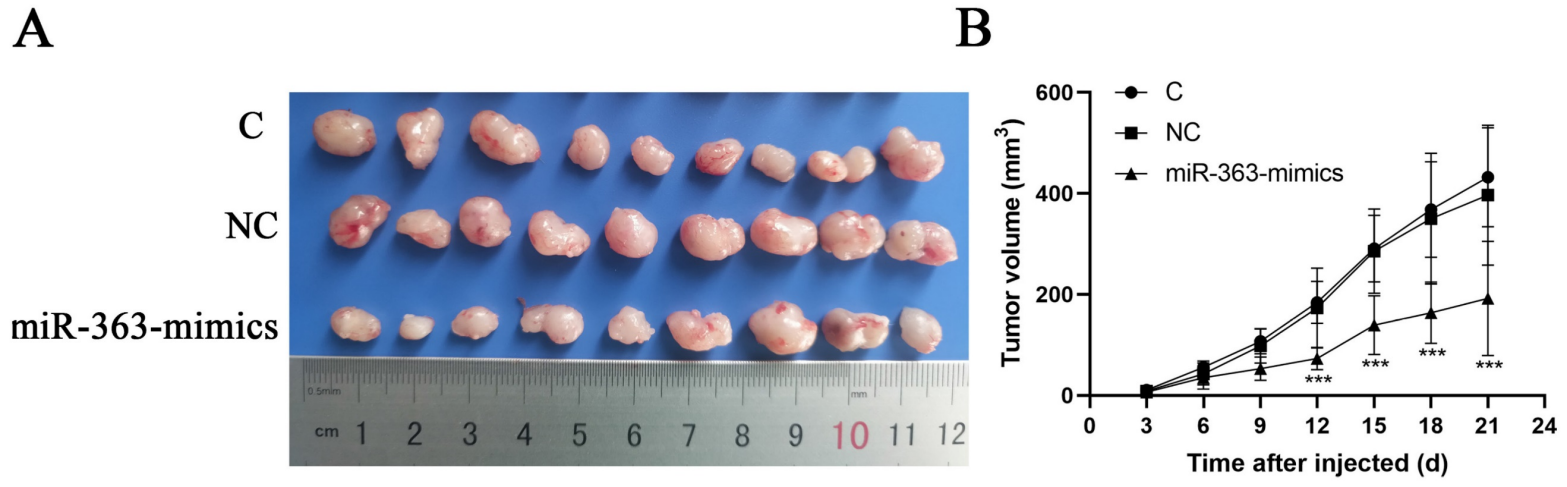
** miR-363 suppresses tumor growth *in vivo*.** A. Images of tumors from transfected with miR-363 mimics or NC after 21 days of injected. B. Volumes of tumors *in vivo* were detected every 3 days. n = 3. ^**^P<0.01, ^***^P<0.001.

HCT-116 cells transfected with miR-363 mimics, NC or C were subcutaneously injected into either flank of nude mice. Compared with NC, miR-363 reduced the volume of tumors formed from HCT-116 cells at different time-points (P<0.001) (Fig 5A and B). The results suggested that the miR-363 might reduce tumorigenicity and tumor progression in the nude mouse model.

## Discussion

Up to now, treatment failure and recurrence of CRC are still an urgent problem to be solved in clinical, and one of the main reasons for the poor prognosis of CRC patients is the late diagnosis 32, 33. Therefore, it is of great significance to find predictive bio-markers.

Numerous studies demonstrated that miRNAs are important in regulating tumor cell proliferation, migration and invasion 34-37, and are involved in the pathogenesis of CRC 38, 39. MiR-4323 promotes CRC cell proliferation by inhibiting HDGF 40. MiR‑4306 inhibits proliferation, migration and invasion by targeting lncRNA-FoxD2‑AS1 in CRC 41. miR-545 promotes colorectal cancer by inhibiting transferring in the non-normal ferroptosis signaling 42. Fusobacterium nucleatum promotes colorectal cancer metastasis through miR-1322/CCL20 axis and M2 polarization 43.

MiR-363 is downregulated in various tumors and regulates tumor cell development 44, 45. Studies reported that 46-48 miR-363 overexpression can significantly reduce the resistance of HepG2-R cells to cisplatin, decrease the migration ability of head and neck tumor cells, and inhibit the renal cancer cell proliferation and migration. Jamali Z et al 49 observed that patients with miR-363 low expression in head and neck tumors had a poor prognosis, and miR-363 role is via targeting of FBW7 ubiquitin ligase expression 50. Aberrant miR-363 expression affects head and neck cancer infiltrate and metastasize by targeting podoplanin 45, indicating that miR-363 plays a role in the development and prognosis of tumors. miR-363 can degrade target genes by fully binding to the 3'UTR region of target genes, or incompletely binding to inhibit the translation process, forming a negative regulatory role. However, the effect of miR-363 on CRC and its mechanism remain to be further studied.

In this study, we first preformed RT-PCR in CRC cell lines and found that miR-363 level in HCT-116 and SW480 cells were markedly lower than that in NCM460 cells. Then, miR-363 mimics were transferred into HCT-116 and SW480 cells, and CCK8, wound-healing assay and cell invasion assay were used to detect cell behavior. The results showed that miR-363 overexpression inhibited CRC cell proliferation, migration and invasion. Cell cycle is divided into the early stage of DNA synthesis (G1 stage), the DNA synthesis stage (S stage) and the late DNA synthesis stage (G2 stage). The main function of G1 phase is to synthesize RNA and ribosomes to prepare material and energy for DNA replication in S phase, S phase is mainly for DNA synthesis, and G2 phase is to prepare for mitosis. Cell cycle experiments showed that miR-363-mimics blocked the cell cycle of HCT-116 and SW480 in G1 phase, suggesting that miR-363-mimics can inhibit DNA synthesis. We further used flow cytometry to detect the cell apoptosis of HCT-116 and SW480. The results showed that the apoptotic rate of HCT-116 and SW480 was increased in miR-363-mimics group compared to the control and NC group, suggesting that overexpression of miR-363 can promote CRC cell apoptosis and inhibit cell proliferation, migration and invasion of CRC.

MMPs is a highly conserved class of enzymes composed of a series of zinc ion dependent proteolytic enzymes, which are widely distributed in various organisms. Under normal physiological conditions, MMPs and tissue metalloproteinases inhibitors (TIMP) co-regulates the renewal of ECM and maintain cell stability, while the imbalance of MMPs can destroy tissue barrier, promote matrix degradation, and then promote tumor invasion and metastasis. According to the substrate and fragment homology, MMPs can be divided into four categories. Gelatinase (i.e. type IV collagenase) is an important class, mainly including MMP9 and MMP2. Studies have shown that gelatinases exhibit increased activity in a variety of malignant tissues, cultured tumor cells, and oncogene transformed cells, and both *in vivo* and *in vitro* invasion assays have confirmed that the high invasive ability of tumor cells is associated with the increased activity of gelatinases, which are thus considered to be the major proteolytic enzymes involved in tumor invasion and metastasis. MMP2 and MMP9 have a lot of the same components in the degradation substrates, but MMP9 has no ability to hydrolyze collagen directly 51. Under physiological conditions, MMP2 is ubiquitously expressed *in vivo*, while MMP9 is only present in neutrophil granules. Numerous studies have demonstrated that MMP-2 plays a critical role in tumor cell-mediated extracellular matrix degradation. The increase of MMP-2 activity and expression is closely related to the invasion and metastasis potential and prognosis of a variety of human malignant tumors 52, so it has become the hotspot of tumor invasion and metastasis research in recent years. Tumor invasion and metastasis is a continuous multi-step process. In addition to the degradation of extracellular matrix, the formation of tumor microvessels is also indispensable for tumor invasion and metastasis 53. MMP2 has the ability to degrade collagen in vascular basement membrane, which is closely related to tumor angiogenesis 54, and its activation state plays a crucial role in angiogenesis. Thus, in this study, we selected MMP2, one of the genes most closely related to tumor invasion and metastasis, for detection, and the results showed that the expression of MMP2 was significantly decreased in the miR-363-mimics group compared with the control and NC groups, and the results still supported the foregoing conclusion.

Luciferase reporter assay was used to test whether E2F3 is a direct target of miR-363. RT-PCR and WB were used to detect the E2F3 level in HCT-116 and SW480 cell which miR-363 overexpressed, and the results showed that E2F3 was target of miR-363. To further confirm the hypothesis that overexpression of miR-363 suppresses cell behavior by targeting E2F3 in CRC, siRNA-E2F3 was transfected to HCT-116 and SW480 cells, and the results indicated that knockdown of E2F3 dramatically inhibited the cell migration and invasion in HCT-116 and SW480 cells.

We inoculated HCT-116 cells transfected with miR-363 mimics into nude mice to observe whether miR-363 could inhibit tumor progression. Results suggested that the miR-363 might reduce tumorigenicity and tumor progression in a nude mouse model, which further confirmed that miR-363 might suppress cell proliferation, migration and invasion by targeting E2F3 in CRC.

In this study, we only used miR-363 mimics to intervene, and observed CRC cell growth and motility. Lacking miR-363 inhibitor is one of the shortcomings of this study, and we will refine it in the next in-depth study.

In conclusion, our research showed that miR-363 was significantly decreased in CRC cells, and miR-363 suppresses cell proliferation, migration and invasion by targeting E2F3 in CRC cells.

## Figures and Tables

**Table 1:** Primer sequences

Primer	Sequence (5'-3')
miR-363-F	ACACTCCAGCTGGGAATTGCACGGTATCCA
miR-363-R	TGGTGTCGTGGAGTCG
E2F3-F	GTATGATACGTCTCTTGGTCTGC
E2F3-R	CAAATCCAATACCCCATCGGG
U6-F	CTCGCTTCGGCAGCACA
U6-R	AACGCTTCACGAATTTGCG
GAPDH-F	ATTCCATGGCACCGTCAAGGCTGA
GAPDH-R	TTCTCCATGGTGGTGAAGACGCCA
